# Safety and effectiveness of mirabegron in male patients with overactive bladder with or without benign prostatic hyperplasia: A Japanese post‐marketing study

**DOI:** 10.1111/luts.12335

**Published:** 2020-08-05

**Authors:** Satoru Takahashi, Daisuke Kato, Hiromi Tabuchi, Satoshi Uno

**Affiliations:** ^1^ Department of Urology Nihon University Tokyo Japan; ^2^ Medical Science, Medical Affairs Astellas Pharma Inc. Tokyo Japan; ^3^ Japan‐Asia Data Science, Development Astellas Pharma Inc. Tokyo Japan

**Keywords:** benign prostatic hyperplasia, effectiveness, mirabegron, overactive bladder, safety

## Abstract

**Objectives:**

The aim of this post hoc analysis from the Japanese mirabegron surveillance program was to investigate the safety and effectiveness of mirabegron in male patients with overactive bladder (OAB) symptoms with/without concomitant benign prostatic hyperplasia (BPH).

**Methods:**

This 12‐week study included patients who were starting mirabegron treatment for the OAB symptoms of urinary urgency, daytime frequency, and urgency urinary incontinence. Patients were stratified according to BPH diagnosis, and patients with BPH were stratified into those who did/did not receive BPH‐specific treatment. Assessments included adverse drug reactions (ADRs), residual urine volume evaluations, and total Overactive Bladder Symptom Score (OABSS) and International Prostate Symptom Score‐Quality of Life (IPSS‐QoL) measurements.

**Results:**

Of 4540 male patients, 3176 (70.0%) had been diagnosed with BPH. Mean age was slightly higher in patients with BPH (74.7 ± 8.41 years) versus patients without BPH (71.0 ± 12.13 years). Overall, 66/1364 (4.84%), 170/2588 (6.57%), and 35/569 (6.15%) patients without BPH, with BPH + treatment, and with BPH + no treatment, respectively, experienced ≥1 ADR. No patients without BPH and 21/3176 (0.66%) patients with BPH experienced a urinary retention ADR. No significant changes from baseline to week 12 in residual volume were noted. Mirabegron was judged to be an effective treatment for 990/1296 (76.4%) patients without BPH, 1935/2491 (77.7%) patients with BPH + treatment, and 421/538 (78.3%) patients with BPH + no treatment. Significant decreases in total OABSS and IPSS‐QoL were observed for all groups.

**Conclusions:**

Mirabegron was a well‐tolerated and effective treatment for patients with OAB symptoms with or without BPH in this post‐marketing study.

## 1 INTRODUCTION

The occurrence of overactive bladder (OAB) syndrome can have a substantial impact on patients' quality of life (QoL), including affecting work productivity, anxiety, and increased healthcare usage.^1^, ^2^ OAB is a prevalent condition, and two epidemiological studies have estimated that 12% of the Japanese population exhibit OAB symptoms.^3^, ^4^ In both of these surveys, a slightly higher prevalence was noted for men compared with women. Patients with severe benign prostatic hyperplasia (BPH) typically experience higher levels of pain, more anxiety, and greater negative effects on their usual activities than patients with milder symptoms.^5^ The coexistence of BPH with OAB could therefore have a substantial impact on the QoL of men with both conditions.^6^

Several pharmacological approaches are recommended for treating patients with BPH and OAB symptoms. Current Japanese guidelines recommend various pharmacological agents, including α_1_‐adrenoreceptor antagonists (α_1_‐blockers; such as tamsulosin), phosphodiesterase 5 inhibitors (such as tadalafil), and 5α‐reductase inhibitors (such as dutasteride) for treating patients with BPH.^7^ In terms of OAB symptoms, potential treatment options in Japan include antimuscarinics or the β3‐adrenoreceptor agonists, mirabegron and vibegron.^7^, ^8^, ^9^ Antimuscarinics are associated with specific anticholinergic side effects, including dry mouth and constipation,^10^ and the β3‐adrenoreceptor agonists may circumvent these effects through their distinct mechanism of action.^11^, ^12^, ^13^

A few initial clinical studies have been conducted that have examined the safety and effectiveness of mirabegron in male patients with OAB symptoms and BPH. The MATCH and PLUS studies both showed that add‐on mirabegron treatment was more effective than add‐on placebo in men with residual OAB symptoms who were receiving tamsulosin for lower urinary tract symptoms (LUTS).^14^, ^15^ An integrated analysis of five phase 3 trials found that similar improvements in urgency, frequency, and incontinence were observed with mirabegron and the antimuscarinic solifenacin in a population of male patients with OAB symptoms.^16^ In the same analysis, approximately 37% of the population had a history of LUTS associated with BPH or benign prostatic enlargement and 22% were receiving treatment with an α_1_‐blocker.^16^

The present study was conducted to assess the safety, effectiveness, and appropriate use of mirabegron in patients with OAB symptoms in the routine clinical environment.^17^ The primary analysis from this study found that mirabegron was a well‐tolerated and effective treatment for Japanese patients with OAB symptoms in the real‐world setting. Using the findings from this study, this post hoc analysis was conducted to examine the safety and effectiveness of mirabegron in male patients with OAB symptoms with or without BPH. In particular, the occurrences of urinary retention and the concomitant use of α_1_‐blockers were specifically investigated.

## 2 METHODS

This study (ClinicalTrials.gov: NCT01919047) is one of the post‐marketing surveys that encompass the mirabegron surveillance program^18^, ^19^ and was planned and conducted in accordance with the Good Post‐Marketing Study Practice (GPSP) standards of the Japanese Ministry of Health, Labour, and Welfare (MHLW).^20^

### 2.1 Study design

The methodology for this study has been reported previously.^17^ In summary, the overall study included male and female patients who were diagnosed with OAB by their attending physician. Eligible patients were receiving their first administration of mirabegron as a treatment for the OAB symptoms of urinary urgency, daytime frequency, and urgency urinary incontinence. The instigation of mirabegron treatment was at the physician's discretion.

Patients were diagnosed with BPH by their attending physician, and no specific criteria had to be met to confirm a positive diagnosis. Male patients were stratified on the basis of this diagnosis in the present subanalysis. This methodology was adopted because this investigation was a large noninterventional study within the clinical setting that included data from multiple healthcare facilities. The patients who had been diagnosed with BPH were further stratified into two groups, those who did or did not receive treatment for BPH during the study. BPH treatment was defined as the prescription of an α_1_‐blocker and/or 5α‐reductase inhibitor, and the use of treatment was at the discretion of the attending physician.

The internet‐based post‐marketing survey data collection system, PostMaNet, was used to register patients and collect data. Physicians were required to register each patient within 14 days of the start of mirabegron treatment. The registered patients were observed for a 12‐week period, and the survey data were entered at the end of this observation period or at discontinuation.

### 2.2 Study assessments

Patient characteristic data were captured prior to the onset of mirabegron treatment, and concomitant medication usage was acquired during the observation period.

In terms of safety evaluations, the incidence of adverse drug reactions (ADRs), which included abnormal findings from laboratory or other tests, was evaluated throughout the study period. All ADRs were summarized and coded using the Japanese Medical Dictionary for Regulatory Activities (MedDRA; version 17.1). An ADR was defined as an adverse event (AE) that was judged by the physicians to be either potentially related to mirabegron or had an unknown relationship with mirabegron. As the physicians may not have reported all the events that were unrelated to mirabegron treatment in this observational study, ADRs were analyzed rather than AEs. In addition, residual urine volume assessments were conducted at baseline and after 12 weeks of mirabegron treatment (or at discontinuation).

The effectiveness assessments included total Overactive Bladder Symptom Score (OABSS) and International Prostate Symptom Score‐Quality of Life (IPSS‐QoL) evaluations, which were completed at baseline and after 12 weeks of mirabegron treatment (or at discontinuation). Changes in OAB symptoms were assessed after 12 weeks (or at discontinuation) and compared with baseline symptoms. The general efficacy of mirabegron treatment was subsequently judged to be “effective,” “ineffective,” or “not evaluable” according to the discretion of the attending physician. An improvement in total OABSS of ≥3 points from baseline was defined as a minimal clinically important change (MCIC).^21^

### 2.3 Statistical analyses

All statistical analyses were conducted using SAS version 9.2 or higher (SAS Institute, Cary, North Carolina). To ensure the detection of ADRs occurring at a low frequency, the planned sample size for the overall study was 10 000 patients.^17^

In terms of analysis sets, the safety analysis set consisted of patients who had no registration infringements, received mirabegron, were willing to confirm the existence of any ADR, and had ≥1 study visit after the initial receipt of medication within the required contract period. Patients included in the efficacy analysis set had been diagnosed with OAB and were eligible for efficacy assessment according to the attending physicians. Of these, patients who did not have major diseases or conditions that excluded a diagnosis of OAB (abnormal bladder, perivesical abnormalities, prostatic or urethral abnormalities, urinary tract or genital infections, urinary retention, polyuria, or psychogenic pollakiuria), were judged to have OAB based on the OABSS (question 3 score [urgency]: ≥2 points at baseline, total OABSS: ≥3 points at baseline), received mirabegron in accordance with the dosing regimen, and had OABSS results at baseline and week 12 (or at discontinuation) were included in the OABSS analysis set.

The Wilcoxon signed rank test was used to assess the changes from baseline to week 12 in residual urine volume, total OABSS, and IPSS‐QoL.

## 3 RESULTS

### 3.1 Study population

The study was conducted from April 2012 to July 2014. Of the 9795 patients included in the overall study, 4588 (46.8%) patients were male.^17^ The presence or absence of concomitant diseases was unknown for 48 patients, and therefore 4540 male patients were included in the safety analysis set for this study. Of these, 3176 (70.0%) patients had been diagnosed with BPH. In total, 4343 patients were included in the efficacy analysis set and 1784 were included in the OABSS analysis set.

The demographic data showed that mean age was slightly higher in the patients with BPH (mean ± standard deviation [SD]: 74.7 ± 8.41 years) compared with the patients without BPH (71.0 ± 12.13 years; Table 1). Mean body mass index was similar in both groups (patients without BPH: 23.03 ± 3.255 kg/m^2^, patients with BPH: 23.16 ± 3.359 kg/m^2^), as was the proportion of patients who had “wet” OAB disease (patients without BPH: 775/1364 [56.8%] patients, patients with BPH: 1806/3176 [56.9%] patients). At baseline, both mean residual urine volume and mean prostate volume were higher in patients with BPH (residual urine volume: 26.075 ± 33.2619 mL, prostate volume: 32.055 ± 17.5525 mL) compared with patients without BPH (residual urine volume: 16.178 ± 22.3285 mL, prostate volume: 16.222 ± 9.0763 mL). In particular, the patients with BPH who received treatment tended to have a higher residual volume (26.872 ± 34.2008 mL) in comparison with the patients with BPH who received no treatment (22.594 ± 28.2540 mL).

**TABLE 1:** Patient demographics and baseline characteristics

Variable	OAB patients without BPH (N = 1364)	OAB patients with BPH			
		Total (N *=* 3176*)*	Received treatment for BPH (N *=* 2588)^a^	Received no treatment for BPH (N *=* 569*)*	Unknown BPH treatment status (N *=* 19*)*
Age in years, mean ± SD	71.0 ± 12.13	74.7 ± 8.41	74.7 ± 8.23	74.4 ± 9.23	79.5 ± 5.82
BMI in kg/m^2^, mean ± SD [n]	23.03 ± 3.255 [601]	23.16 ± 3.359 [1367]	23.18 ± 3.404 [1105]	23.08 ± 3.127 [253]	22.64 ± 4.337 [9]
OAB disease classification, n (%)					
Dry	362 (26.5)	912 (28.7)	762 (29.4)	146 (25.7)	4 (21.1)
Wet	775 (56.8)	1806 (56.9)	1467 (56.7)	327 (57.5)	12 (63.2)
Unknown	227 (16.6)	458 (14.4)	359 (13.9)	96 (16.9)	3 (15.8)
Residual urine volume in mL, mean ± SD [n]	16.178 ± 22.3285 [904]	26.075 ± 33.2619 [2212]	26.872 ± 34.2008 [1803]	22.594 ± 28.2540 [397]	21.600 ± 38.1876 [12]
Prostate volume in mL, mean ± SD [n]	16.222 ± 9.0763 [717]	32.055 ± 17.5525 [2405]	32.220 ± 17.7028 [1956]	31.081 ± 16.8689 [434]	38.581 ± 16.1597 [15]
Concomitant drugs during the observation period, n (%)					
No	781 (57.3)	326 (10.3)	0	326 (57.3)	NK
Yes	536 (39.3)	2831 (89.1)	2588 (100)	243 (42.7)	NK
α_1_‐blocker and/or 5α‐reductase inhibitor	51 (3.7)	2588 (81.5)	2588 (100)	0	NK
α_1_‐blocker	50 (3.7)	2521 (79.4)	2521 (97.4)	0	NK
5α‐reductase inhibitor	2 (0.1)	325 (10.2)	325 (12.6)	0	NK
Antimuscarinic	80 (5.9)	207 (6.5)	167 (6.5)	40 (7.0)	NK
Unknown	47 (3.4)	19 (0.6)	0	0	19 (100)
Concomitant diseases, n (%)					
No	567 (41.6)	0	0	0	0
Yes (≥5.0% of patients in either OAB patients without BPH or OAB patients with BPH [total] groups)	797 (58.4)	3176 (100)	2588 (100)	569 (100)	19 (100)
Prostatic hyperplasia	0	3176 (100)	2588 (100)	569 (100)	19 (100)
Hypertension	372 (27.3)	1099 (34.6)	893 (34.5)	200 (35.1)	6 (31.6)
Diabetes mellitus	141 (10.3)	367 (11.6)	306 (11.8)	59 (10.4)	2 (10.5)
Hyperlipidemia	94 (6.9)	227 (7.1)	178 (6.9)	49 (8.6)	0
Prostate cancer	146 (10.7)	146 (4.6)	101 (3.9)	44 (7.7)	1 (5.3)

*Note*: Data are shown for the safety analysis set.

Abbreviations: BMI, body mass index; BPH, benign prostatic hyperplasia; NK, not known; OAB, overactive bladder; SD, standard deviation.

^a^ Patients received treatment with an α_1_‐blocker and/or 5α‐reductase inhibitor.

Out of the patients with BPH, 2588/3176 (81.5%) received treatment for BPH during the study and 569 (17.9%) did not receive any treatment (unknown status: 19 patients). In total, 2521 (79.4%) of the patients with BPH were receiving treatment with an α_1_‐blocker and 325 (10.2%) patients were receiving treatment with a 5α‐reductase inhibitor. Overall, 287/4540 (6.3%) of the patients in the entire patient population received treatment with an antimuscarinic during the observation period. In total, 51 (3.7%) OAB patients without BPH received an α_1_‐blocker and/or 5α‐reductase inhibitor. From the data available, it was not possible to accurately ascertain why the patients without BPH may have been receiving an α_1_‐blocker and/or 5α‐reductase inhibitor for reasons not related to the treatment of BPH. However, data were obtained that showed that the α_1_‐blockers urapidil and terazosin were used by five patients and one patient for the treatment of neurogenic bladder and hypertension, respectively.

Concomitant diseases were noted in 797/1364 (58.4%) of the patients without BPH. Major concomitant diseases in this population were hypertension (372 [27.3%] patients), prostate cancer (146 [10.7%] patients), and diabetes mellitus (141 [10.3%] patients). Other than prostatic hyperplasia, the major concomitant diseases in the patients with BPH were hypertension (1099/3176 [34.6%] patients), diabetes mellitus (367 [11.6%] patients), and hyperlipidemia (227 [7.1%] patients).

### 3.2 Safety

Slightly higher ADR incidences were observed for the patients with BPH compared with the patients without BPH (Table 2). In total, 66/1364 (4.84%), 170/2588 (6.57%), and 35/569 (6.15%) patients without BPH, with BPH and received treatment, and with BPH and received no treatment, respectively, experienced ≥1 ADR during the study.

**TABLE 2:** Occurrence of ADRs

Variable	OAB patients without BPH	OAB patients with BPH			
		Total	Received treatment for BPH^a^	Received no treatment for BPH	Unknown BPH treatment status
Total patients, n	1364	3176	2588	569	19
Patients with ADRs, n	66	206	170	35	1
ADRs, n	74	231	191	38	2
Proportion of patients with ADRs, % of total patients	4.84	6.49	6.57	6.15	5.26
Lower urinary tract obstruction‐related ADRs, n (% of total patients)					
Residual urine volume increased	8 (0.59)	39 (1.23)	34 (1.31)	5 (0.88)	0
Dysuria	4 (0.29)	24 (0.76)	20 (0.77)	4 (0.70)	0
Urinary retention	0	21 (0.66)	16 (0.62)	5 (0.88)	0

*Note*: Data are shown for the safety analysis set. Data shown in the table are reproduced from “Safety and effectiveness of mirabegron in patients with overactive bladder in a real‐world clinical setting: a Japanese post‐marketing study” by Nozawa Y et al, which is licensed under CC BY 4.0.

Abbreviations: ADR, adverse drug reaction; BPH, benign prostatic hyperplasia; CC BY, Creative Commons Attribution; OAB, overactive bladder.

^a^ Patients received treatment with an α_1_‐blocker and/or 5α‐reductase inhibitor.

The incidences of lower urinary tract obstruction‐related ADRs were slightly higher in patients with BPH compared with the patients without BPH (Table 2). For example, residual urine volume increased was noted in 8/1364 (0.59%) patients without BPH, 34/2588 (1.31%) patients with BPH who received treatment, and 5/569 (0.88%) patients with BPH who received no treatment.

In terms of the ADR of urinary retention specifically, none of the patients without BPH experienced an ADR, whereas 21/3176 (0.66%) patients with BPH experienced urinary retention. The majority of these patients were ≥ 75 years old (15/21 [71.4%] patients) and received BPH treatment (16 [76.2%] patients; Table S1). Treatment with mirabegron was discontinued due to urinary retention for 19 patients, discontinued in one patient upon request, and continued in one patient. The outcome was determined to be “resolved” or “recovering” for 19 patients and “unknown” for two patients. In total, 16 (76.2%) of the patients who experienced an ADR of urinary retention were receiving concomitant treatment with mirabegron and an α_1_‐blocker and six (28.6%) patients were receiving concomitant treatment with an antimuscarinic.

**FIGURE 1:**
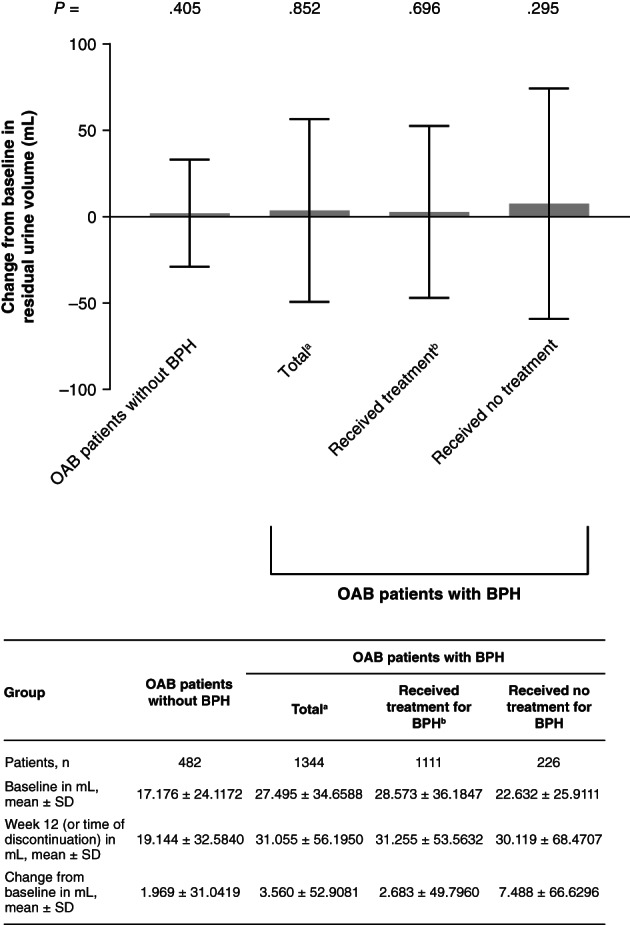
Changes from baseline to week 12 (or time of discontinuation) in residual urine volume. Data are shown for the safety analysis set. Results are expressed in terms of mean ± SD. *P* values were derived using the Wilcoxon signed rank test. ^a^Includes data from seven patients with unknown BPH treatment status. ^b^Patients received treatment with an α_1_‐blocker and/or 5α‐reductase inhibitor. BPH, benign prostatic hyperplasia; OAB, overactive bladder; SD, standard deviation

Mean residual urine volume was higher at baseline in patients with BPH (mean ± SD—received treatment: 28.573 ± 36.1847 mL, received no treatment: 22.632 ± 25.9111 mL) compared with patients without BPH (17.176 ± 24.1172 mL). No statistically significant changes from baseline in residual volume were noted during the study. However, higher increases were noted for the patients with BPH, particularly for the group that did not receive any treatment (received treatment: 2.683 ± 49.7960 mL, received no treatment: 7.488 ± 66.6296 mL), compared with those who had not been diagnosed with BPH (1.969 ± 31.0419 mL; Figure 1).

### 3.3 Effectiveness

As judged by the physicians, mirabegron was considered to be an effective treatment for similar proportions of the patients with and without BPH (without BPH: 990/1296 [76.4%] patients, with BPH and received treatment: 1935/2491 [77.7%] patients, with BPH and received no treatment: 421/538 [78.3%] patients).

**FIGURE 2:**
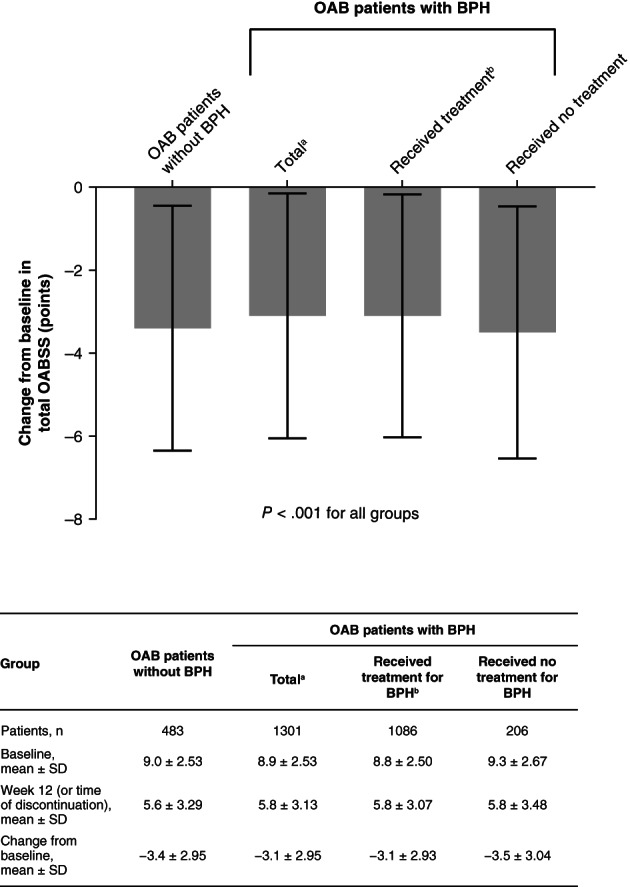
Changes from baseline to week 12 (or time of discontinuation) in total OABSS. Data are shown for the OABSS analysis set. Results are expressed in terms of mean ± SD. *P* values were derived using the Wilcoxon signed rank test. ^a^Includes data from nine patients with unknown BPH treatment status. ^b^Patients received treatment with an α_1_‐blocker and/or 5α‐reductase inhibitor. BPH, benign prostatic hyperplasia; OAB, overactive bladder; OABSS, Overactive Bladder Symptom Score; SD, standard deviation

Marginally higher mean changes from baseline in total OABSS were noted for the patients without BPH (mean ± SD: −3.4 ± 2.95) and the patients with BPH who received no treatment (−3.5 ± 3.04) compared with the patients with BPH who received treatment (−3.1 ± 2.93; Figure 2). However, significant decreases in total OABSS were observed for all of the groups (*P* < .001), and all of the mean decreases observed satisfied the criteria for an MCIC. The proportion of patients who achieved an MCIC in total OABSS was slightly lower for the patients with BPH who received treatment (601/1086 [55.3%] patients) compared with the patients without BPH (304/483 [62.9%] patients) and the patients with BPH who received no treatment (126/206 [61.2%] patients). Significant decreases were also observed for each of the groups when the results were stratified in terms of each individual question from the OABSS (Figure S1).

**FIGURE 3:**
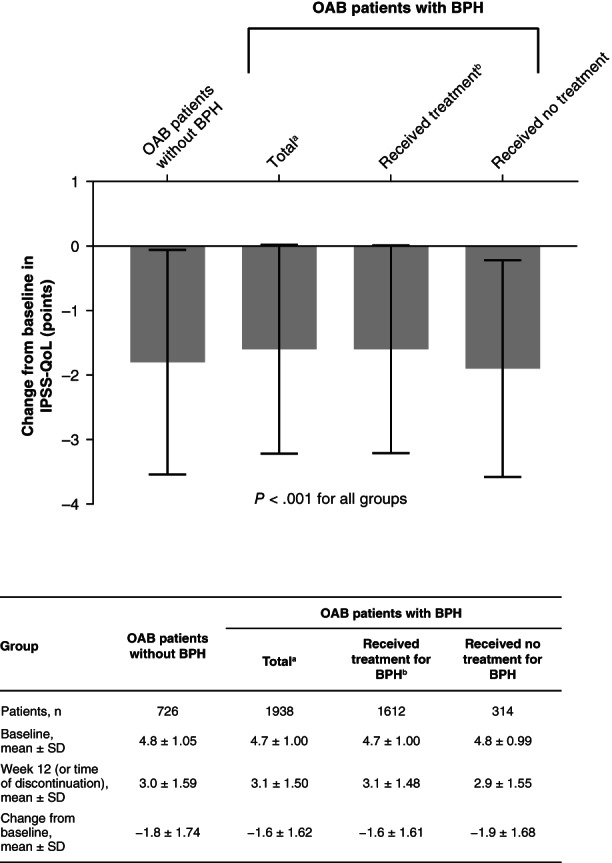
Changes from baseline to week 12 (or time of discontinuation) in IPSS‐QoL. Data are shown for the efficacy analysis set. Results are expressed in terms of mean ± SD. *P* values were derived using the Wilcoxon signed rank test. ^a^Includes data from 12 patients with unknown BPH treatment status. ^b^Patients received treatment with an α_1_‐blocker and/or 5α‐reductase inhibitor. BPH, benign prostatic hyperplasia; IPSS‐QoL, International Prostate Symptom Score‐Quality of Life; OAB, overactive bladder; SD, standard deviation

Significant decreases from baseline were observed for all of the groups in terms of IPSS‐QoL (*P* < .001; Figure 3). Slightly lower mean changes from baseline in IPSS‐QoL were noted for the patients with BPH who received treatment (mean ± SD: −1.6 ± 1.61) compared with the patients without BPH (−1.8 ± 1.74) and the patients with BPH who received no treatment (−1.9 ± 1.68).

## 4 DISCUSSION

The mirabegron surveillance program in Japan has substantially contributed to the quantity of data available that demonstrate the efficacy and safety of mirabegron in the clinical setting.^17^, ^18^, ^19^ Although clinical trials have been conducted that have analyzed the efficacy and safety of mirabegron add‐on therapy for treating OAB symptoms in men concurrently receiving tamsulosin for LUTS,^14^, ^15^ this is the first post‐marketing study that indicates that mirabegron can be an effective treatment for OAB symptoms regardless of whether the patient has concomitant BPH. If a patient has concomitant OAB and BPH, physicians need to monitor the patient for increased residual urine, especially if they are receiving a medication that may increase their risk.

Overall ADR incidences were slightly higher for the patients with BPH compared with those without BPH. This finding was attributed to the higher occurrence of lower urinary tract obstruction‐related ADRs in patients with BPH. Overall, a low incidence of urinary retention (21/4540 [0.46%] patients) was noted in this study. This finding supports the results of previous investigations where urinary retention was reported with an incidence of 0.24% (two patients) in a pooled analysis that investigated the efficacy and safety of mirabegron in male patients with OAB symptoms.^16^ Furthermore, no cases of urinary retention were noted in the MATCH study in which men with OAB received either add‐on mirabegron or placebo in conjunction with an α_1_‐blocker, tamsulosin, for the treatment of LUTS.^14^ Only one case of urinary retention was observed following the administration of mirabegron add‐on treatment to 43 patients with OAB symptoms and benign prostatic obstruction who were receiving tamsulosin treatment.^22^

Owing to the low incidence of lower urinary tract obstruction‐related ADRs noted here, no additional concerns have arisen from this study that require the instigation of further safety measures. However, mirabegron should be administered with caution to patients with clinically significant bladder outlet obstruction,^23^ and patients with OAB symptoms and BPH who are receiving the drug should be monitored for signs of urinary retention.

Although slightly higher increases in mean residual volume were observed for the patients with BPH, no statistically significant changes from baseline were noted in this study. However, the highest increases were noted for patients who did not receive treatment, which indicates that the use of BPH treatment may help alleviate any increases in residual volume. The fact that mirabegron was not associated with clinically relevant changes in residual volume is an important observation given that the medication is associated with a lower incidence of urinary retention compared with antimuscarinics.^24^ In support of the mirabegron findings from this study, previous studies involving male patients with OAB symptoms have not typically reported any substantial changes in post‐void residual volume over the mirabegron treatment period.^14^, ^16^, ^25^

The overall effectiveness findings from this study showed that mirabegron was a successful treatment for OAB symptoms. Mirabegron was considered to be an effective treatment for 77.4% of the male patients with OAB who participated in this study, and a very similar result (77.8%) has been reported in a 3‐year post‐marketing study that is also part of the mirabegron surveillance program.^19^

In this study, the use of mirabegron was associated with significant decreases in both total OABSS and IPSS‐QoL. Similar findings have been observed in previous investigations. Compared with placebo, mirabegron administration was associated with greater changes from baseline in total OABSS and IPSS subscores in the MIRACLE study which enrolled 464 males with OAB symptoms.^26^ Similarly, in MATCH, statistically significant reductions in total OABSS and IPSS (including IPSS‐QoL) were reported with mirabegron compared with placebo in men receiving concurrent treatment with tamsulosin.^14^ In addition, similar overall MCIC results (defined as an improvement in total OABSS of ≥3 points) of 58.1% and 65.1% have been respectively observed in our study and the 3‐year mirabegron surveillance study.^19^ In contrast to the above results, no significant difference was noted in total IPSS between the tamsulosin plus mirabegron and the tamsulosin plus placebo groups in the PLUS study.^15^

One of the strengths of this study is the high number of patients who were involved in this surveillance. The overall study included approximately 10 000 patients^17^ and over 4500 male patients were included in this substudy. In addition, owing to the varied population involved, this study more accurately reflects the real‐world situation compared with a clinical trial population, where specific inclusion and exclusion criteria have to be satisfied for enrollment. Furthermore, we believe that the results of this study are clinically meaningful and help demonstrate the effectiveness of mirabegron in real‐world practice. A limitation of this study is that no placebo or active control arms were included in this real‐world surveillance. In addition, the fact that no diagnostic criteria had to be satisfied to confirm the diagnosis of BPH and that the condition was diagnosed solely according to the physician's judgment is a potential limitation. Furthermore, potential bias due to unadjusted or unmeasured confounding factors caused by the observational nature of the study design should be noted.

In conclusion, this post‐marketing study showed that mirabegron is a well‐tolerated and effective treatment for patients with OAB symptoms with or without BPH. When considering both safety and effectiveness, combination therapy with BPH treatment and mirabegron could therefore be considered as a potential therapeutic option in the clinical setting for patients with OAB symptoms and concomitant BPH.

## DATA SHARING STATEMENT

Researchers may request access to anonymized participant level data, trial level data, and protocols from Astellas sponsored clinical trials at www.clinicalstudydatarequest.com.

For the Astellas criteria on data sharing see: https://clinicalstudydatarequest.com/Study-Sponsors/Study-Sponsors-Astellas.aspx.

## CONFLICT OF INTEREST

Satoru Takahashi has received consultancy, lectureship, and advisory board member fees and nonfinancial support from Astellas Pharma Inc. and consultancy and lectureship fees from Pfizer, Nihon Shinyaku, Kyorin, Kissei, Daiichi Sankyo, Taiho, and Hisamitsu. Daisuke Kato, Hiromi Tabuchi, and Satoshi Uno are all employees of Astellas Pharma Inc.

## Supporting information


**FIGURE S1.** Changes from baseline to week 12 (or time of discontinuation) in OABSS questions: question 1 (a), question 2 (b), question 3 (c), and question 4 (d)

**TABLE S1.** List of patients who experienced an ADR of urinary retention
