# Supplementary material for: Safety and effectiveness of mirabegron in male patients with overactive bladder with or without benign prostatic hyperplasia: A Japanese post‐marketing study
Source: Low Urin Tract Symptoms. 2020 Aug 5;13(1):79–87. doi: 10.1111/luts.12335 (PMC7818393; doi:10.1111/luts.12335)
Supplement: Supplementary file 1 — FIGURE S1. Changes from baseline to week 12 (or time of discontinuation) in OABSS questions: question 1 (a), question 2 (b), question 3 (c), and question 4 (d) [file LUTS-13-79-s001.pdf]

FIGURE S1 Changes from baseline to week 12 (or time of discontinuation) in OABSS questions: question 1 (a), question 2 (b), question 3 (c), and question 4 (d)

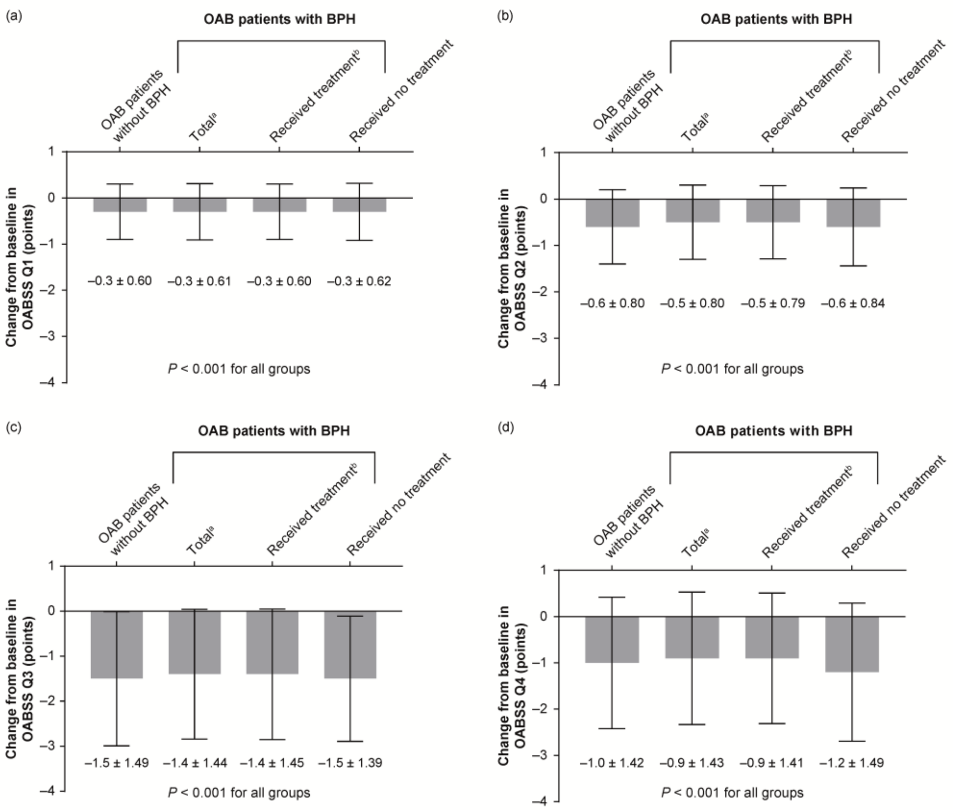

Data are shown for the OABSS analysis set. Results expressed in terms of mean ± SD. *P* values were derived using the Wilcoxon signed rank test.
[a]Includes data from nine patients with unknown BPH treatment status.
[b]Patients received treatment with an α_1-blocker and/or 5α-reductase inhibitor.
Abbreviations: BPH, benign prostatic hyperplasia; OAB, overactive bladder; OABSS, Overactive Bladder Symptom Score; SD, standard deviation.
