# Supplementary material for: Safety and effectiveness of mirabegron in male patients with overactive bladder with or without benign prostatic hyperplasia: A Japanese post‐marketing study
Source: Low Urin Tract Symptoms. 2020 Aug 5;13(1):79–87. doi: 10.1111/luts.12335 (PMC7818393; doi:10.1111/luts.12335)
Supplement: Supplementary file 2 — TABLE S1. List of patients who experienced an ADR of urinary retention [file LUTS-13-79-s002.docx]

TABLE S1 List of patients who experienced an ADR of urinary retention

| **Serial number** | **ADR** | **Age in years** | **Prostate volume at baseline in mL** | **Concomitant drugs^a^** | **Residual urine volume at baseline in mL** | **Residual urine volume at ADR onset in mL** | **Mirabegron** | | | **α_1_-blocker** | | **5α-reductase inhibitor** | | **Antimuscarinic** | | **Date of ADR onset** | **Mirabegron treatment** | **Outcome** | **Serious** | **Causal relationship** |
| --- | --- | --- | --- | --- | --- | --- | --- | --- | --- | --- | --- | --- | --- | --- | --- | --- | --- | --- | --- | --- |
|  |  |  |  |  |  |  | **Start date** | **Stop date** | **Days of treatment** | **Start date** | **Stop date** | **Start date** | **Stop date** | **Start date** | **Stop date** |  |  |  |  |  |
| 00197-005 | Urinary retention | 69 | 31 | 1, 4 | NR | NR | 16 Nov 12 | 25 Feb 13 | 102 | Before mirabegron | 28 Mar 13 | – | – | – | – | 24 Feb 13 | Discontinued | Resolved | No | Possible |
| 00202-022 | Urinary retention | 72 | – | 1, 2 | 54 | 900 | 12 Nov 12 | 19 Dec 12 | 38 | Before mirabegron | 19 Dec 12 | – | – | Before mirabegron | 19 Dec 12 | 19 Dec 12 | Discontinued | Resolved | No | Unlikely |
| 00260-003 | Residual urine | 81 | 30 | 1 | 0 | 100 | 13 Nov 12 | 13 Feb 13 | 93 | Before mirabegron | Continued | – | – | – | – | 13 Feb 13 | Discontinued | Unknown | No | Possible |
| 00260-009 | Residual urine | 74 | 20 | 1 | 0 | 100 | 04 Dec 12 | 05 Mar 13 | 92 | Before mirabegron | Continued | – | – | – | – | 05 Mar 13 | Discontinued | Unknown | No | Possible |
| 00439-006 | Urinary retention | 79 | 55 | 1, 4 | NR | NR | 08 Dec 12 | 15 Jan 13 | 39 | Before mirabegron | 15 Jan 13 | – | – | – | – | 15 Jan 13 | Discontinued | Resolved | No | Possible |
| 00459-017 | Urinary retention | 81 | 95 | 1, 4 | 28 | NR | 05 Jan 13 | 17 Jan 13 | 13 | Before mirabegron | 15 Jan 13 | – | – | – | – | 17 Jan 13 | Discontinued | Resolved | No | Possible |
| 00475-008 | Urinary retention | 83 | 20.04 | 1, 3 | 0 | NR | 18 Jul 12 | 10 Sep 12 | 55 | Before mirabegron | Continued | Before mirabegron | Continued | – | – | 10 Sep 12 | Discontinued | Resolved | No | Unlikely |
| 00495-014 | Urinary retention | 81 | 50 | 1, 2, 3, 4 | NR | 800 | 14 Aug 12 | 07 Sep 12 | 25 | Before mirabegron | Continued | Before mirabegron | Continued | Before mirabegron | 07 Sep 12 | 07 Sep 12 | Discontinued | Recovering | No | Possible |
| 00537-003 | Urinary retention | 79 | – | 1, 2 | 60 | NR | 13 Jun 13 | 27 Jun 13 | 15 | Before mirabegron | Continued | – | – | Before mirabegron | 27 Jun 13 | 27 Jun 13 | Discontinued | Recovering | Yes | Possible |
| 00584-010 | Feeling of residual urine | 71 | 36 | 1, 2 | 23 | 83 | 20 Jul 12 | 17 Aug 12 | 29 | Before mirabegron | Continued | – | – | Before mirabegron | Continued | 17 Aug 12 | Discontinued upon patient request | Resolved | No | Unknown |
| 00657-009 | Urinary retention | 70 | 35 | 1 | 0 | NR | 31 May 13 | 05 Jul 13 | 36 | Before mirabegron | Continued | – | – | – | – | 05 Jul 13 | Discontinued | Resolved | No | Unknown |
| 00667-005 | Urinary retention | 77 | 22 | 1, 4 | NR | 300 | 19 May 12 | 25 May 12 | 7 | Before mirabegron | Continued | – | – | – | – | 24 May 12 | Discontinued | Recovering | No | Possible |
| 00754-007 | Urinary retention | 81 | – | 1 | 83 | 600 | 23 Oct 12 | 04 Jan 13 | 74 | Before mirabegron | Continued | – | – | – | – | 04 Jan 13 | Discontinued | Resolved | No | Unknown |
| 00768-001 | Urinary retention | 78 | – | 0 | 35 | NR | 05 Jul 12 | 07 Sep 12 | 65 | – | – | – | – | – | – | 07 Sep 12 | Discontinued | Resolved | No | Possible |
| 00792-006 | Urinary retention | 76 | 64 | 1, 2 | NR | 590 | 07 Nov 12 | 05 Dec 12 | 29 | Before mirabegron | Continued | – | – | Before mirabegron | Continued | 05 Dec 12 | Discontinued | Recovering | No | Possible |
| 00828-010 | Residual urine | 81 | 28 | 0 | 80 | 211 | 25 Jan 13 | 07 Feb 13 | 14 | – | – | – | – | – | – | 07 Feb 13 | Discontinued | Resolved | No | Possible |
| 00865-005 | Residual urine | 81 | – | 1 | 20 | 116 | 14 Sep 12 | Continued | N/A | Before mirabegron | Continued | – | – | – | – | 20 Oct 12 | Continued | Recovering | No | Possible |
| 00945-020 | Urinary retention | 74 | 20 | 0 | 20 | NR | 26 Jul 12 | 23 Aug 12 | 29 | – | – | – | – | – | – | 23 Aug 12 | Discontinued | Resolved | No | Possible |
| 00968-002 | Urinary retention | 77 | 104 | 1, 3, 4 | 53 | 671 | 19 Sep 12 | 21 Sep 12 | 3 | Before mirabegron | Continued | Before mirabegron | Continued | – | – | 21 Sep 12 | Discontinued | Recovering | No | Possible |
| 00995-001 | Urinary retention | 83 | 26 | 2 | 24 | 850 | 13 Jun 12 | 09 Jul 12 | 27 | – | – | – | – | Before mirabegron | 13 Jul 12 | 08 Jul 12 | Discontinued | Resolved | No | Possible |
| 01140-002 | Urinary retention | 83 | 23 | 0 | NR | 620 | 09 Aug 12 | 13 Aug 12 | 5 | – | – | – | – | – | – | 12 Aug 12 | Discontinued | Resolved | No | Possible |

^a^Concomitant drugs key: 0, none; 1, α_1_-blocker; 2, antimuscarinic; 3, 5α-reductase inhibitor; 4, others.
Abbreviations: ADR, adverse drug reaction; N/A, not applicable; NR, not recorded.
